# Post-fire structural forest recovery associated with climate extremes in dry sub-boreal forests

**DOI:** 10.1007/s10980-025-02266-y

**Published:** 2025-12-01

**Authors:** Sarah Smith-Tripp, Nicholas Coops, Christopher Mulverhill, Joanne White, Sarah Gergel

**Affiliations:** 1 Department of Forest Resources Management, Faculty of Forestry, University of British Columbia, 2424 Main Mall, Vancouver, BC Canada; 2 Canadian Forest Service, Pacific Forestry Centre, 506 West Burnside Road, Victoria, Natural Resources Canada V8Z 1M5 Canada; 3 Present Address: Department of Forest and Conservation Sciences, Faculty of Forestry, University of British Columbia, 3024-2424 Main Mall, Vancouver, British Columbia V6T 1Z4 Canada

**Keywords:** Post-fire regeneration, Climate extremes, Ecological drivers, Landscape dynamics, Remote sensing, Ecological resilience

## Abstract

**Context:**

Recent large and high-severity wildfires have burned vast areas of coniferous forests throughout Western North America. These burned landscapes are recovering amid increasingly frequent climate extremes, such as drought. We need to understand how post-fire climate extremes and other ecological drivers (such as fire impacts) influence patterns and trends of coniferous recovery.

**Objectives:**

We worked at a landscape scale (> 400,000 hectares) to investigate the association between distinct post-fire forest recovery and ecological drivers in dry sub-boreal forests. We created structural recovery groups distinct in patterns and trends of coniferous cover and density and then modeled their association with ecological drivers.

**Methods:**

We used Landsat time-series data to identify unique spectral recovery, which we grouped based on post-fire regrowth and stocking estimates. Remotely Piloted Aircraft light detection and ranging (lidar) provided structural estimates 5–21 years post-fire. We modeled the association between structural recovery groups and ecological drivers with random forests. For each category of drivers (site conditions, climate, climate anomalies, pre-fire composition, and fire impacts), we used individual models to identify important drivers. We then incorporated the most important drivers in a global model to highlight the drivers that were important across categories.

**Results:**

Initial spectral trends indicated longer-term differences in structural forest recovery. Climate anomalies (such as post-fire extremes in temperature and precipitation) and pre-fire basal area best predicted observed structural groupings—abnormally cold and dry summers after the fire were associated with slow conifer establishment. Comparatively, areas with a higher pre-fire basal area maintained a mixed canopy of deciduous and coniferous stems.

**Conclusions:**

At a landscape scale, post-fire climate conditions best predicted structural forest recovery, suggesting management plans should be adaptable to the conditions experienced post fire.

**Supplementary Information:**

The online version contains supplementary material available at 10.1007/s10980-025-02266-y.

## Introduction

Global increases in twenty-first century fire severity and intensity (Pausas and Keeley 2021) are accentuated in forests of Western North America (NA, Abatzoglou et al. 2021; Parisien et al. 2023). In the province of British Columbia (BC), the last decade has seen multiple years of record-breaking wildfire seasons, linked both to historic fire suppression (Baron et al. 2022) and increasingly common hot and dry summers (Hanes et al. 2019; Parisien et al. 2023). Forests are not only more likely to burn because of climate change (Hanes et al. 2019), but also recover amidst a warmer and drier climate (Davis et al. 2019).

Historically, Western NA forests were conifer-dominant (Hessburg et al. 2005), but altered fire dynamics alongside a changing climate could promote failed conifer regeneration (Prichard et al. 2021). Distinct post-fire conifer growth, henceforth structural recovery, describes recovering forests that vary in conifer composition, structure, and growth rates. Post-fire seed availability, from the soil or remaining canopy, is a prerequisite for conifer establishment (Turner et al. 1999; Stewart et al. 2021). Ecological drivers, including site conditions or legacies of the fire event control seed germination and germinant growth. Variability in the structural recovery of conifers is then linked with spatial and temporal variation in ecological drivers. These drivers can be split into categories that include: fire impacts (Taylor et al. 2021), site-level topography (Peeler and Smithwick 2021), pre-fire vegetation conditions (White et al. 2023), climatic regime (Chu and Guo 2014), and post-fire climate conditions (Davis et al. 2023).

Fire impacts, such as remaining post-fire canopy, can alter seed availability, germination, and post-fire microclimate (Turner et al. 1999; Peeler and Smithwick 2021; Smith-Tripp et al. 2022). Remaining post-fire forest canopy can also protect conifer germinants from climate extremes (Davis et al. 2023) and fire can catalyze seedling germination in serotinous lodgepole pine (*Pinus contorta var. latifolia*) populations (Lotan 1976). Yet the importance of fire impacts is variable. For example, serotiny in lodgepole pine is more common at high elevations and in the north (Lotan 1976). Comparably, environmental and site-level conditions, such as elevation or slope, alter conifer recovery independent of species (Stevens-Rumann and Morgan 2019). Across the Greater Yellowstone Ecosystem, Kiel and Turner (2022) found that higher elevations and steeper slopes caused sparse conifer growth (< 1,000 stems/ha) more than 30 years post-fire. Yet, such relationships are not ubiquitous: in the Blue Mountains of Oregon, Downing et al. (2019) found a positive relationship with elevation and conifer seedling density.

Region and site history drive pre-fire vegetation conditions, which in turn impact conifer recovery. A high density and/or basal area of conifers pre-fire can increase the rate and density of conifer recovery (Harvey et al. 2016; White et al. 2023). Additionally, a higher pre-fire deciduous composition can result in rapid deciduous growth post-fire (Haire and McGarigal 2008). Post-fire deciduous growth can facilitate or compete with conifer growth—Downing et al. (2019) found deciduous growth negatively impacted recovery of most conifers but protected young ponderosa pines (*Pinus ponderosa*) from climate extremes. Finally, disturbance histories, including consecutive burns or insect attacks, can dramatically alter recovery patterns. Short intervals between burns can limit available serotinous seeds because the trees that establish after the first burn do not reach sexual maturity before the second burn (Braziunas et al. 2023). Other disturbances, such as Western Canada’s infamous mountain pine beetle (MPB) infestation, can cause canopy death, introducing additional fuel for fire (Talucci et al. 2022), as well as decreasing the available seed stock (Teste et al. 2011).

Pine forests throughout BC were decimated by MPB, raising important questions on how the legacy of the MPB impacts both the likelihood of fire and recovery post-fire. Starting in the early 2000s, the MPB outbreak impacted over half (50%) of mature lodgepole pine stands (Dhar et al. 2016b). Many researchers expected that the aftermath of MPB would be extensive areas of tree mortality, which would be ideal fuel for high-intensity and high-severity fire (Collins et al. 2012). However, observed outcomes of MPB and fire are more mixed (Harvey et al. 2014). In the early years after initial MPB attack, fire susceptibility increases, but in later years, the likelihood of fire is lower (Harvey et al. 2013). Yet, as MPB-affected trees die and fall to the ground, they leave behind large fuel that, when ignited, burn the soil at high severities (Perrakis et al. 2018; Talucci et al. 2022). Given the expansive area impacted by MPB, it is crucial to consider how combined disturbance of MPB and fire could impact ecosystem recovery (Dhar et al. 2018).

In conifer forests south of BC, recent models of post-fire recovery heavily emphasize the importance of extreme post-fire climate, such as drought or extreme heat on conifer recovery ( Vanderhoof et al. 2021: Stevens-Rumann et al. 2022; ). For example, Talucci et al. (2019) studied lodgepole pine recruitment following wildfire and bark beetle infestation in interior BC. They found that post-fire climate moisture deficit (CMD) was negatively correlated with seedling density, but the sample size-limited study conclusions. Across the Western US region, Davis et al. (2023) combined observations from > 10,000 field plot samples to model post-fire recruitment probability. In their models, a year of extremely hot and dry conditions negatively impacted conifer recruitment, but impacts varied across scales, regions, and species (Davis et al. 2023). The importance of post-fire climate conditions has been demonstrated in dry forests of the Western US (Guz et al. 2021; Stewart et al. 2021) and in boreal environments (Boucher et al. 2020; White et al. 2023), but investigations of post-fire climate, relative to other drivers, are lacking in the dry sub-boreal region of interior BC.

To understand recovery drivers, landscape-level approaches are helpful to capture a gradient of ecological drivers. Exemplars such as the meta-analysis of Davis et al. (2023) help clarify the impact of different drivers on ecosystem recovery. However, building such landscape-scale field datasets is costly, labour-intensive, and challenging to update. Literature syntheses, comparing individual study results from a diversity of environments, are another way to understand recovery drivers across scales (Bartels et al. 2016). However, BC’s sub-boreal region, despite encompassing over 12 million hectares, has had relatively few post-fire studies and lacks the extensive field sampling used in other meta-analyses (e.g., Davis et al. 2023). The lack of post-fire monitoring and research in the extensively burned sub-boreal (Parisien et al. 2023) could preclude us from capturing possibly novel landscape transitions – such as conifer forests transitioning to conifer grasslands (Hamilton and Burton 2023).

Satellite imagery is a strategic way to overcome the data deficits in BC’s under-sampled sub-boreal forests (Chu and Guo 2014). Long-term and historically continuous satellite measures, such as the 40-year Landsat archive, capture temporal trends in surface reflectance and associated land cover (White 2024). Spectral indices, calculated from surface reflectance, describe different elements of forest structure. The rates of spectral recovery for different indices, like the normalized burn ratio (NBR), capture structural forest development, including patterns of stem density or time-points of conifer establishment (Kiel and Turner 2022; Smith-Tripp et al. 2024a; White et al. 2023). Using early spectral recovery trends (e.g. five years), descriptive of longer (e.g. 20 years) distinct structural recovery, helps infer how recently burned environments may structurally recover in the future (Pickett 1989; Pettorelli et al. 2005). In this case, future recovery rates, both structural and spectral, could be inferred by observations in older burns with similar early spectral responses (Ye et al. 2021; Smith-Tripp et al. 2024b).

In the face of widespread fires, landscape-level forest structure monitoring can leverage the Landsat archive as well as the spatially continuous structural measures from light detection and ranging data (lidar) to identify how early spectral responses are attributed to structural development over time (Menick et al. 2024). Even early spectral responses (< 5 years) have been linked to longer-term differences in post-fire structural growth (Smith-Tripp et al. 2024b). Lidar captures spatial patterns of recovery at the scale necessary to link structural estimates to satellite-based temporal trends, and how these patterns and trends vary based on ecological drivers (Weiss et al. 2023).

Our study capitalizes on the link between spectral responses and structural recovery (Smith-Tripp et al. 2024b) to investigate the relative impact of recovery drivers at a landscape scale. We ask which ecosystem drivers are associated with different patterns and trends of structural recovery. Our objectives were three-fold: (1) develop structural groupings that define forest structural recovery in relation to spectral recovery responses (2) determine the relative effect that different categories of drivers (e.g. post-fire climate or pre-fire conditions) have on structural groupings; and (3) across all recovery drivers, understand which drivers were the most important across and within structural groupings. Our approach highlights recovery drivers with the greatest impact on post-fire structural patterns and trends in the western sub-boreal forests of British Columbia.

## Methods

### Study region

**Fig. 1:**
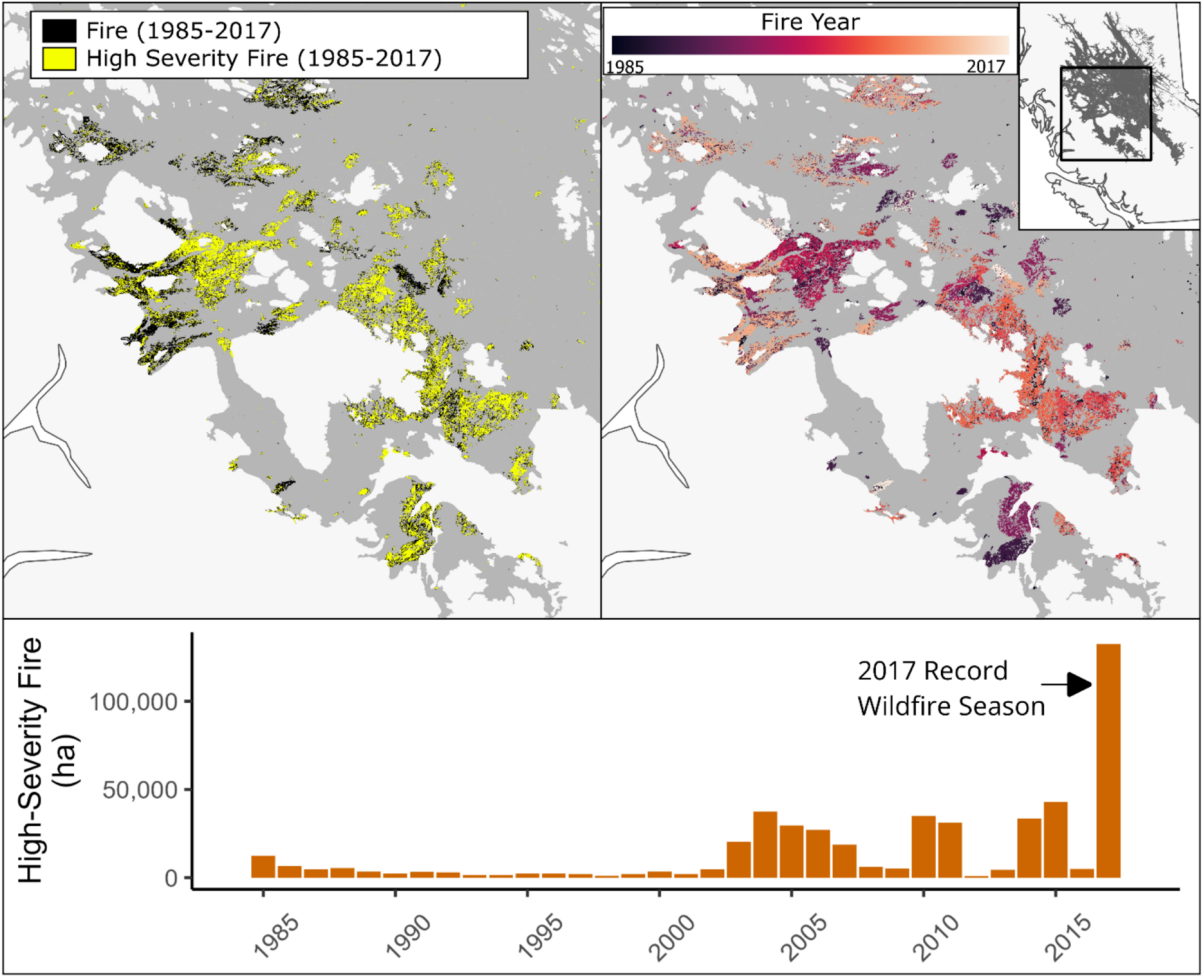
Burned areas included in research. (Top left) NTEMS identified fires (yellow) and areas above high-severity threshold (red) based on change magnitude. (Top right) Years of fire event from 1985–2017. Inset map at top right notes location or study area within British Columbia. (Bottom) column chart displaying hectares (ha) of high-severity fire for all study years. The 2017 record wildfire season is highlighted. Total area of fires = 430,000 ha

Our study area encompassed over 300 fires that burned nearly 500,000 ha of forest between 1985 and 2017 in central British Columbia, Canada (Fig. 1, CWFIS). We concentrated on the sub-boreal spruce (SBS) and sub-boreal pine spruce (SBPS) biogeoclimatic ecozones. The SBS and SBPS ecoregions cover 12 million hectares of rolling topography and high-elevation plateaus (600 – 1300 m elevation). The climate is continental with dry and cool growing seasons; temperatures and precipitation average 12.3 ˚C and 166 mm, respectively (Wang et al. 2016). Soils are typically podzols or luvisols of glacial and glaciofluvial origin (DeLong et al. 2003). The historic fire regime in these ecozones was large stand-replacing fires that occurred every 100 years, but recent fire events were larger and more severe than historic norms (Pelletier et al. 2024). After these stand-replacing burns, similar to other sub-alpine and sub-boreal North American forests, post-fire regrowth consists of rapid lodgepole pine (*Pinus contorta var. latifolia)* canopy establishment alongside resprouts of deciduous species such as trembling aspen (*Populus tremuloides*) and paper birch (*Betula papyrifera*; Day 1972; Pausas and Keeley 2021). Many areas of the SBS and SBPS are single-species stands of lodgepole that were established after the last fire event (Meidinger and Pojar 1991). Other conifer species typically establish beneath this lodgepole pine canopy. These species include: spruce (including Engelman, white, and their hybrid, *Picea engelmannii, glauca,* and *engelmannii x glauca*)*,* Douglas-fir (*Pseudotsuga menziezii*), subalpine fir *(Abies lasiocarpa*; DeLong et al. 2003).

### Methods overview

**Fig. 2:**
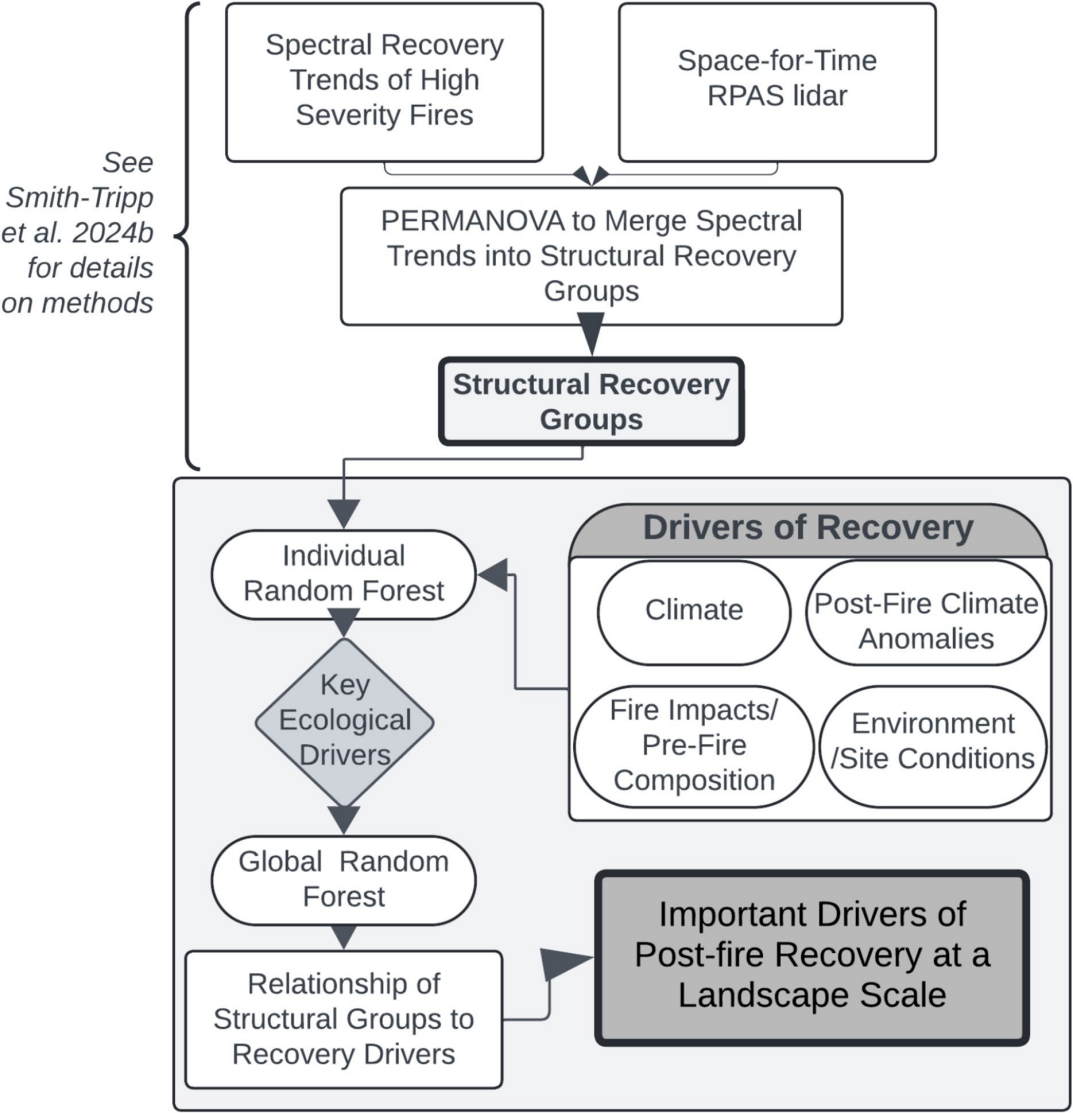
Overall research framework including development of structural groupings. Boxes beneath ‘drivers of recovery’ are the categories of drivers used in individual random forests that were then consolidated in a global random forest. The overall impact of drivers of recovery on structural recovery (final research objective) is accented in dark gray

Our research fused spectral data from Landsat satellites with a chronosequence of RPA lidar-derived forest structural data (5–21 years post-fire) to describe forest structural recovery following high severity fire. Building on the link between unique spectral recovery responses and structural recovery (Smith-Tripp et al. 2024a; 2024b) we created post-fire structural recovery groups using a combination of early spectral responses from Landsat and a space-for-time dataset of forest structure from RPA. We investigated how these observed structural groupings were predicted by ecological drivers (climate, post-fire climate anomalies, fire-impacts, and pre-fire landscape characteristics). An overview of our methodology is presented in Fig. 2.

High-severity wildfire areas were identified using Landsat-based National Terrestrial Ecosystem Monitoring System (NTEMS) data (Hermosilla et al. 2016). These data layers use annual NBR measures to generate Canada-wide maps of disturbance year, type, and magnitude from 1985–2017. Change magnitudes are synonymous with the differenced normalized burn ratio (dNBR) calculated as the difference between the NBR value the year before the fire and the lowest NBR value in the subsequent two years post-fire (Hermosilla et al. 2016). We converted NTEMS change magnitude estimates into Burned Area Reflectance Classification – Adjustable (BARC-A) values, which range from 0 to 255. BARC-A values describe the condition of the surface vegetation that remains after a fire (Hudak et al. 2007). The province of BC uses BARC-A values and additional aerial validation to classify unburned, low, moderate, and high severity burned areas (BC Ministry of Forests 2023), but classifications are only available for fires after 2015 (BC Ministry of Forests 2021). As our study covered 1985–2017, we set a minimum BARC-A threshold of 130 for the transformed NTEMs magnitude estimates, constraining research to areas of high and moderate severity (BC Ministry of Forests 2021).

To eliminate differences in post-fire management, we also removed areas reported as replanted (British Columbia Data Catalogue 2022). Noting the expansive area of BC impacted by MPB, we checked for overlap between selected fire events and areas of historic MPB outbreaks. Regions affected by MPB were identified from BC aerial overview survey (AOS) data. As AOS data is known to have a high error rate (Bourgeois et al. 2018), we required disturbance polygons to have contained MPB infestations in multiple years. We then assessed the overlap between MPB-affected forest polygons with areas the NTEMS algorithm identified as experiencing a non-stand-replacing disturbance followed by a fire event (Hermosilla et al. 2019). The total area impacted first by MPB and then a high-severity fire was low (less than 4% of the study area), and deemed inconsequential to study outcomes. Thus, the final study area covered 430,000 ha and is highlighted in Fig. 1.

### Structural recovery groupings

To create structural recovery groups, we first clustered post-fire spectral metrics to identify unique spectral responses. Spectral metrics described the rate and total magnitude of spectral recovery in the first five years post-fire. The three spectral metrics were calculated for seven spectral indices and included (1) regrowth magnitude, (2) median yearly rate of change (slope), and (3) the spectral reflectance measure 5 years after the fire (indicative of the post-fire landcover; Hicke et al. 2003). Thus, 21 post-fire spectral metrics (three metrics for seven spectral indices) were derived from post-fire measures of seven indices. Indices include the normalized difference vegetation index (NDVI), the normalized difference moisture index (NDMI), the normalized burn ratio (NBR), and the tasseled cap indices (brightness – TCB, wetness – TCW, greenness – TCG, and angle – TCA). For more details on how these metrics are calculated see Smith-Tripp et al. (2024b). Final clustering also included burn severity (dNBR). A total of 22 spectral metrics were standardized and used as inputs into an augmented Kmeans +  + algorithm, which iteratively groups pixels by similarity in n-dimensional (*n* = 22) space (Kapoor and Singhal 2017). Final clusters minimized the distortion criterion, which measures the distance between points and the associated centroid (Kodinariya and Makwana 2013). Clusters representing less than 1% of observations were excluded from further processing.

We merged spectral clusters into distinct structural groupings using a space-for-time sample of forest structural measures captured with RPA lidar. The forest structure sampling covered 1300 ha (an additional 500 ha compared to Smith-Tripp et al. (2024b)) of forests 5–21 years post-fire. We combined RPA lidar data and field measurements to model basal area (BA), bare ground (%), stem density (stems/900 m^2^), and the ratio of coniferous to deciduous cover. To improve temporal continuity, we grouped samples into post-fire “epochs” of one-to-two-year periods: 5–7, 8–9, 11–12, 15–16, and 21-years post-fire. For more details on RPA lidar data and associated lidar modelling, Supplemental Sect. 1 as well as Smith-Tripp et al (2024a, b). We used RPA lidar structural estimates to merge unique spectral trends into distinct recovery groups. A distinct group could have different stem counts, but potentially similar BA across years. We selected a PERMANOVA because it can capture dissimilarity across multiple factors and is robust to non-normal data (Anderson 2017). An initial PERMANOVA used in *F*-statistic to test if trends differed based on a dissimilarity matrix derived from estimates of stem densities, bare ground, basal area, and the proportion of conifer to deciduous by sample year (McArdle and Anderson 2001). Then, spectral clusters were merged into structural groups based on post-hoc tests of the PERMANOVA similarity estimates (*p*-value > 0.05; Todorov 2007). Based on these results we assigned structural grouping names that reflected their distinct patterns or trends, such as stem-density differences or the rate of coniferous establishment. For a more in-depth overview of the grouping process, see Smith-Tripp et al. (2024b).

### Drivers of forest recovery

To investigate how recovery drivers impact structural recovery, we considered both individual drivers, such as elevation, and drivers by category, such as environment. Driver categories include: (a) environment (b) pre-fire vegetation condition and fire-impacts (c) climate and (d) post-fire climate anomalies. We selected drivers based on findings in similar ecosystem types. See Table 1 for data sources and literature support for driver selection.

**Table 1:** Data sources or calculations citations for drivers of forest recovery

Type	Explanatory variable	Abbreviation /Unit	Data Source	Data Reference	Driver Reference
Site/Environmental Conditions	Elevation	Elev (m)	Global digital elevation model	Natural Resources Canada, (2013)	Bright et al. (2019)
	Topographic position index	TPI	Elevation data		Littlefield (2019)
	Soil type	Soil	Terrestrial ecosystem mapping	Heung et al. (2022)	Baltzer et al. (2021)
	Flow direction	Flow direction	Elevation data	Kopecký et al. (2021)	Harvey et al. (2016)
	Aspect index	TRASP	Elevation data	Roberts and Cooper (1989)	Littlefield et al. (2016)
	Ecosystem subzone	Subzone	Ecosystem Classification	Meidinger & Pojar (1991)	BC Forest Practices Board (2020)
Pre-Fire Conditions	Pre-fire BA	Pre-fire BA (m^2^/ha)	NTEMS	Matasci et al. 2018	White et al. (2023)
	Pre-fire landcover class	Pre-fire landcover	NTEMS	Hermosilla et al. (2022a, b)	Meng et al. (2015)
	Pre-fire Species type	Pre-fire species	NTEMS	Hermosilla et al. (2022)	
Fire Impacts	Patch size	Patch (ha)	Derived from NTEMS burned area (Hermosilla et al. 2016)		Kemp et al. (2016)
	Distance to live-edge	Edge (m)			Littlefield et al. 2016
Climate *(& Post-fire Climate Anomalies)*	Mean annual temperature	MAT (°C) *warmest year*	ClimateNA	Wang et al. (2016)	Bright et al. (2019)
	Mean annual precipitation	MAP (mm) *driest year*			Bright et al. (2019)
	Precipitation as snow (mm august – july)	PAS (mm) *min snow*			Talucci et al. (2019)
	Hargreaves climatic moisture deficit	CMD (mm) *max CMD*			Davis et al. (2023)
	Summer average temperature	Summer avg. t *average coldest summer T*			Guz et al. (2021)
	Maximum summer temperature	Summer max t *max warmest summer T*			Guz et al. (2021)
	Minimum summer temperature	Summer min T *min coldest summer T*			Hansen and Turner (2019)
	Minimum winter temperature	Winter min t *min coldest winter T*			Meng et al. (2015)
	Average spring precipitation	Spring precip *min spring precip*			Hankin et al. (2019)
	Average summer precipitation	Summer precip *min summer precip*			Guz et al. (2021)
	Average autumn precipitation	*Fall precip* *min fall precip*			Harvey et al. (2016)

Abbreviation and units for each variable. References note data source. For each driver, a reference for both the data source, and prior reference support (last table column). Note: climate anomalies used in models are italicized

#### Environmental conditions

Elevation data used the bare-ground Canadian Digital Elevation Model (CDEM) hosted by the BC data catalogue (GeoBC, 2014; Natural Resources Canada, 2013). We used the elevation model to calculate additional indices including topographic position index (TPI) in 3 × 3 window, flow direction and transformed aspect (TRASP). Flow direction is a unitless index that describes the drainage of an area, indicative of site-level moisture dynamics (Metcalfe et al. 2015). TRASP transforms aspect to range from 0–1, where 0 is on northern aspects and 1 is on hotter southern aspects (Roberts and Cooper 1989). Soil types were obtained from a provincial digital soil map, classified using a random forest algorithm with validation plots and remotely-sensed climate and vegetation data (Heung et al. 2022).

#### Pre-fire conditions and fire impacts

Pre-fire site conditions used provincial data-layers and satellite-based models. Site-type was from the Biogeoclimatic Ecosystem Classification system (BEC) subzone. The BEC subzone describes precipitation and temperature (e.g., moist-cold) of a given site relative to conditions throughout BC (Meidinger and Pojar 1991). Land cover type, species composition, and BA were derived from NTEMS data (Matasci et al. 2018; Hermosilla et al. 2022b, a). For land cover and species composition, we used the most common class five years prior to the fire. For pre-fire BA, we calculated mean BA (m^2^/ha) five years prior to the fire.

Fire impacts were calculated using the NTEMS disturbance attribution data (Hermosilla et al. 2016). Generally, accurate assessment of fire impacts on soil and canopy from satellite data is limited (Chu and Guo 2014). Additionally, while fire severity is an important driver to consider, it was excluded from models because our study focused only on areas of high severity fire and included severity within initial cluster development. We were constrained to impacts such as the distance to patch-edge (m) and patch size (ha). We calculate these metrics by grouping burned pixels for each study-year (i.e., fire events).

#### Climate and post-fire climate anomalies

Climate data were generated from ClimateNA software, which uses elevation models to downscale data from > 10,000 meteorological stations across North America via bilinear interpolation and dynamic local downscaling (Daly et al. 2008; Wang et al. 2016). First, we used our 30 m elevation data and ClimateNA to calculate and downscale 1981–2010 climate normals at seasonal and annual timesteps. Prior research studies informed climate variable selection (Meng et al. 2015; Petrie et al. 2016; Hankin et al. 2019; Bright et al. 2019; Littlefield 2019; BC Forest Practices Board 2020; Hoecker et al. 2020). Climate normals described average annual and growing season aridity and temperatures. We used climate normals to calculate standardized z-scores of climate extremes. Throughout the text, these extremes are referred to as “climate anomalies.” Climate anomalies used the standardized maximum and/or minimum measure of climate variables for the first five years post-fire to the climate normal and the standard deviation for years 1981–2010. In Table 1, selected climate anomalies are included in italics below the climate normal used to calculate them.

### Modeling recovery drivers impact on structural recovery groups.

To investigate the association between recovery drivers and recovery groupings, we used random forests (RF) modeling where the response variable was the structural recovery group. We selected a random forest approach for three key reasons: (1) RFs are robust to overfitting with a large number of ecological predictors, (2) they do not require linearity or independence among predictors (Fox et al. 2017); and (3) they are computationally efficient for large datasets (Wright and Ziegler 2017). For model building, we selected a stratified random sample of 1% of burned pixels (1985–2017; n = 48,766) stratified by the four structural groups. To limit spatial autocorrelation, sampled pixels were a minimum distance 90 m apart, which we split into training (70%) and testing (30%) sets.

Our RF modeling used a tiered approach. First, we built four individual RFs for each driver category (e.g., environmental conditions or climate). We used the accuracy estimates of the individual RFs to test which category of drivers best predicted structural groups. We also used the variable importance of individual RFs to extract the top five drivers for each category. The global RF combined the dominant drivers of each category. In the cases where the categories had fewer than five variables, we included drivers whose permutational variable importance estimate was greater than 0.05. The tiered RF approach eliminated redundant variables. A total of 34 recovery drivers were used in four individual RF models (Table 1), which consolidated to 19 recovery drivers for the global RF. For individual RFs, the number of variables tested in each split (*mtry*) was 2, and the number of decision trees (ntree) was 500.

The global RF model tested five variables at each split (*mtry*), the number of decision trees (*ntree*) was 500, and the minimum terminal node size (*nodesize*) was five. As we modeled classes, the split rule used extra trees, and the performance measure used the misclassification rate. We tested the accuracy of the global RF model based on out-of-bag (OOB) error with a training dataset and using validation data external to the RF model (sample size = 14,440). To understand the importance of recovery drivers within and across structural groups, we calculated conditional variable importance across the global model and for each structural grouping. We ranked important recovery drivers using the Boruta algorithm, which measures the mean importance of all drivers included in the model compared to random noise in the data when fit multiple times (Kursa and Rudnicki 2010). To understand the likelihood of observing structural responses for different values of recovery drivers, we calculated the partial dependence for the six most important drivers by structural group.

All models were built in an R environment (R Core Team 2023). RF modeling used the *ranger* package (Wright and Ziegler 2017). We used the Boruta algorithm from the *Boruta* package to calculate conditional variable importance (Kursa and Rudnicki 2010). Partial dependence was calculated using the *pdp* package (Greenwell 2017).

## Results

### Identifying structural groups

**Fig. 3:**
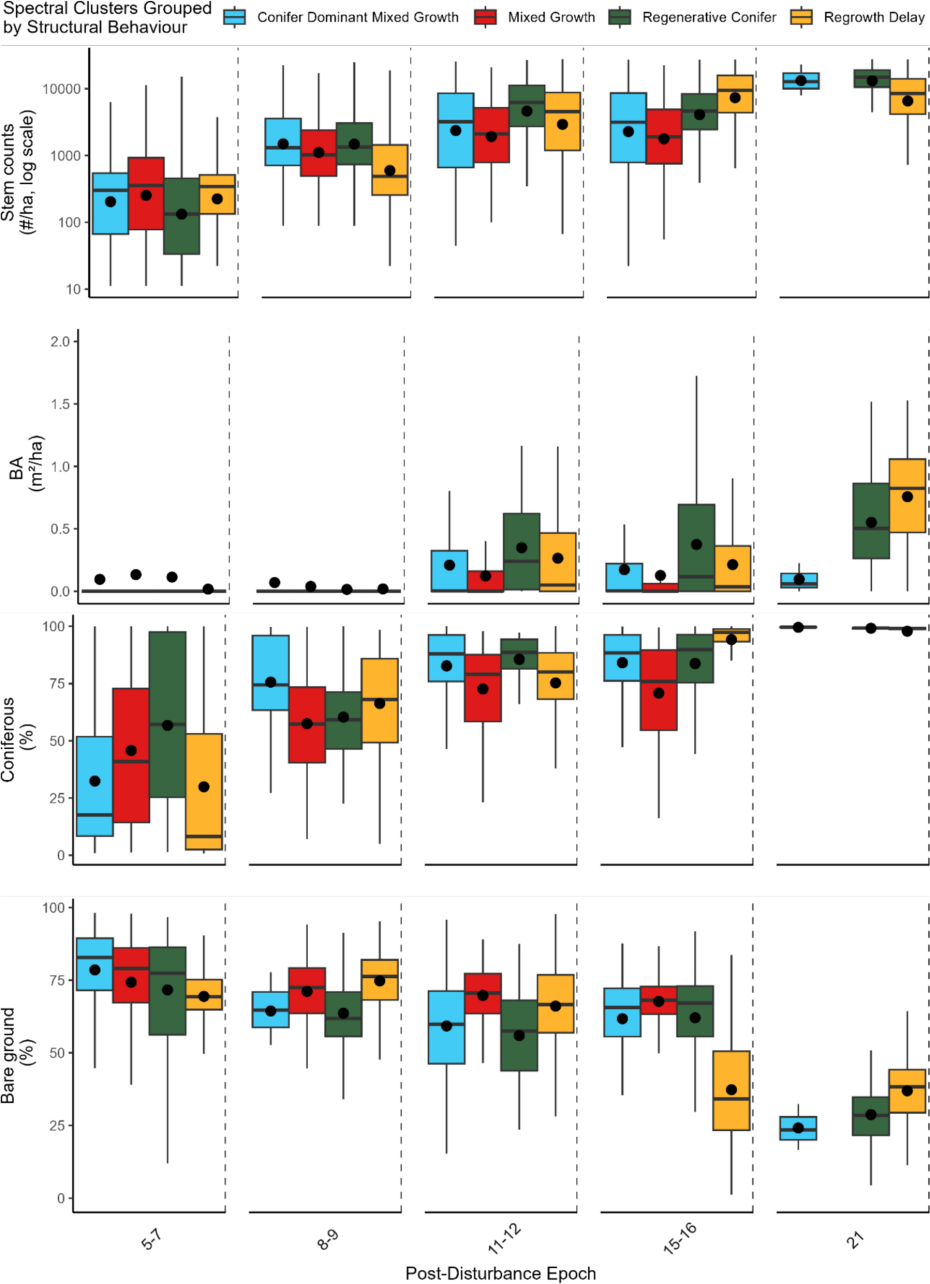
Structural estimates for each post-disturbance epoch by group. Black lines are the median structural value with boxes spanning the interquartile range and 95% confidence values. Black points are the mean structural value. Sample size for calculations is noted in Table S3. Note that mixed growth was not identified in areas sampled 21 years post-fire

Structural groupings described variability in forest structural development through time and across structural variables (Fig. 3). Data-clustering resulted in 8 unique spectral clusters that captured 47% of variance in spectral metrics. For these 8 unique clusters, results of PERMANOVA and post-hoc analysis identified four unique structural groupings within and across years (F (1, 14,385) = 40.62, p < 0.01; Fig S1, Table S2). See Fig. S2 for illustrative orthophotos comparing groups 15 years post-fire. Structural groups were labelled as follows:

*Mixed Growth:* strong deciduous tree and shrub dominance early on (average in year 5–7 post-fire = 48% deciduous) with a low proportion of bare ground in later years (average = 7.9%).

*Regrowth Delay:* a high proportion of bare ground for the first decade (average in years 8–9 = 75%) replaced by dense coniferous stems (average = 10,500 stems/ha in years 15–16).

*Regenerative Conifer:* Strong early coniferous regrowth (average coniferous cover in years 8–9 = 84%) with high-stem densities in later years (highest stem density estimates in year 21 average = 15,035 stems/ha).

*Coniferous Dominant Mixed Growth:* Generally lower stem densities (average in year 15–16 = 5,500 stems/ha) that corresponds with lower BA (average in year 21 = 0.07 m^2^/ha).

**Fig. 4:**
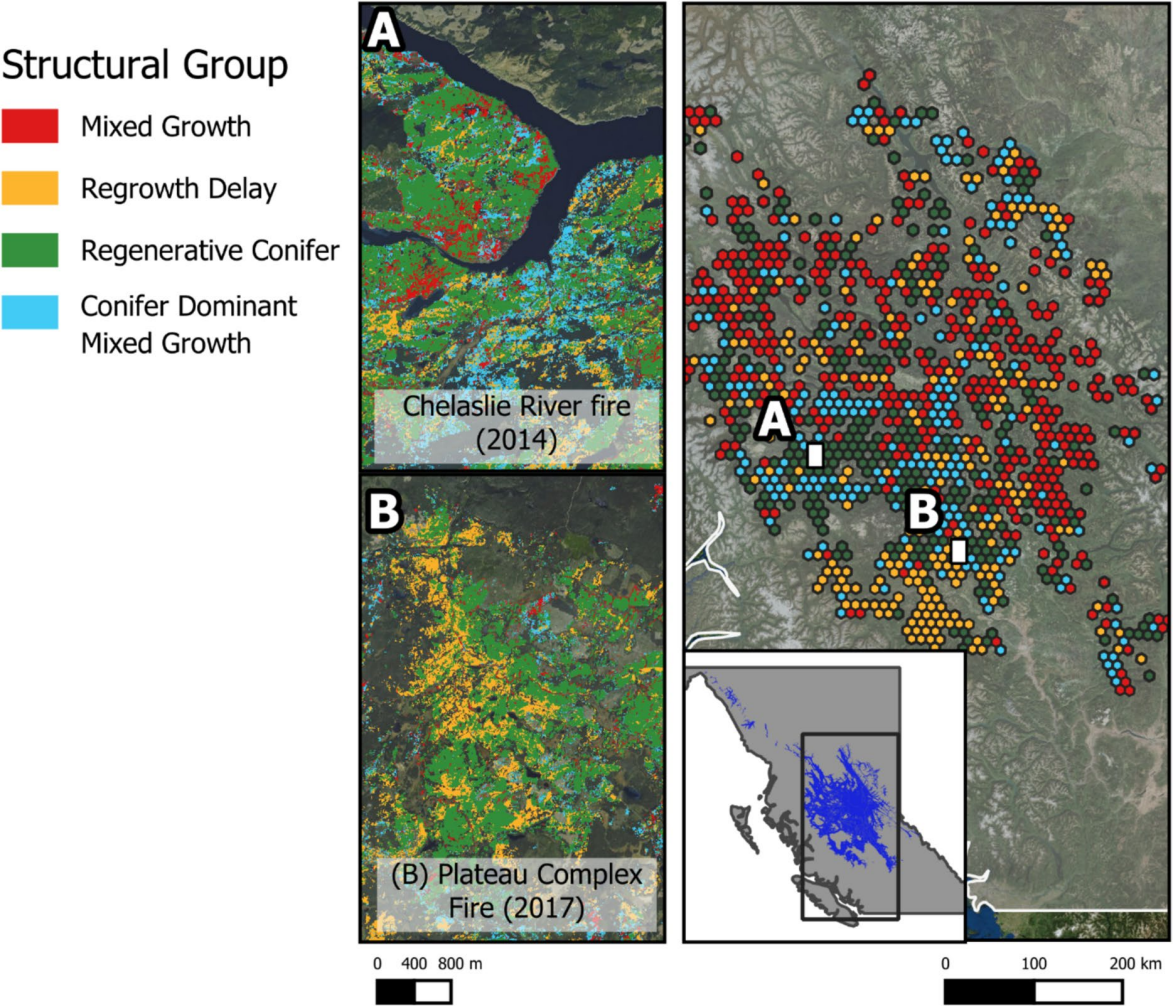
Spatial distribution of structural groupings across the sub-boreal spruce and sub-boreal pine spruce Ecozones – inlay map shows ecozone location within BC. Hexagons are the most common structural group within each 10,000-ha area. Subsets shown left of the map are the groups at a 30 m resolution for two large fires (A—Chelaslie River Fire 2014, B- Plateau Complex Fire 2017). Locations of subset are noted left of map

The most frequently observed grouping was regenerative conifer (38%) followed by regrowth delay (27%), while coniferous dominant mixed growth and mixed growth were less commonly observed (19.7–15.3%). Across the study area, mixed growth and coniferous dominant mixed growth were more common in the north whereas regrowth delay was more common in the south of the study region (Fig. 3). Mixed growth was common in smaller patches – dominant in the northern part of the study region (Fig. 3, Fig. 4,). Regrowth delay and coniferous dominant mixed growth are common in large fires – concentrated in the southwest of the study area.

## Spectral differences among merged structural groups

Recovery groups varied in their scaled spectral recovery metrics used as inputs in k-means clustering. Generally, regrowth delay and conifer dominant mixed growth had lower slopes of spectral recovery (Fig S3). Regrowth delay had the lowest slope of recovery for both TCW and NBR (scaled averages −0.72 and −0.95). Conversely, mixed growth and regenerative conifer had higher slopes of recovery (scaled averages of 0.17 and 0.69 for NBR). Regenerative conifer had the highest slope of recovery for the majority of indices, while mixed growth recovery had the highest slope of recovery for NBR (average = 0.73). Regenerative conifer also had the highest magnitude of regrowth for NBR, NDVI, TCA, TCB, TCG, while mixed growth had the highest regrowth for TCG and NDMI. Comparatively, areas that were identified as conifer dominant mixed growth had the lowest regrowth magnitudes across all indices (Fig S3). However, average spectral measures were similar between conifer mixed growth and regenerative conifer five years after the fire. Finally, burn severity varied across structural groups (H (3, *n* = 39,553) = 9,608.7 (*p* < 0.001)). Conifer dominant mixed growth and regenerative conifer had the greatest burn severities (scaled averages −0.65 and −0.55 respectively). While regrowth delay and mixed growth had lower estimates of burn severity (scaled averages −0.43 and −0.48).

## Structural groupings are predicted by recovery drivers

Following our tiered modeling approach, we first used four individual random forests to assess the predictive power of recovery drivers by category. Individual random forests were built for (1) environment and site conditions, (2) pre-fire conditions and fire impacts, (3) climate conditions, and (4) post-fire climate anomalies. For individual RFs, RFs of post-fire climate anomalies and typical climate conditions had the highest overall accuracy (out-of-bag (OOB) accuracy 83 and 84% respectively, Table S3). Site-level/environmental conditions had an overall accuracy of 54. Finally, pre-fire conditions and fire impacts had an accuracy of 66%. Building on the individual models, the global RF, which used dominant drivers identified for individual models, included 19 predictor variables. The global RF OOB accuracy was 84%, and when tested using an independent validation dataset the overall accuracy was 82%—supporting the model was not overfit. Regenerative conifer was predicted most accurately (mean of precision and recall (F-score) of 89%, Fig. S4), while conifer dominant mixed growth was predicted with the lowest accuracy (F-score = 85%). Both conifer dominant mixed growth and regenerative conifer were frequently misclassified as mixed growth (12% of samples).

**Fig. 5:**
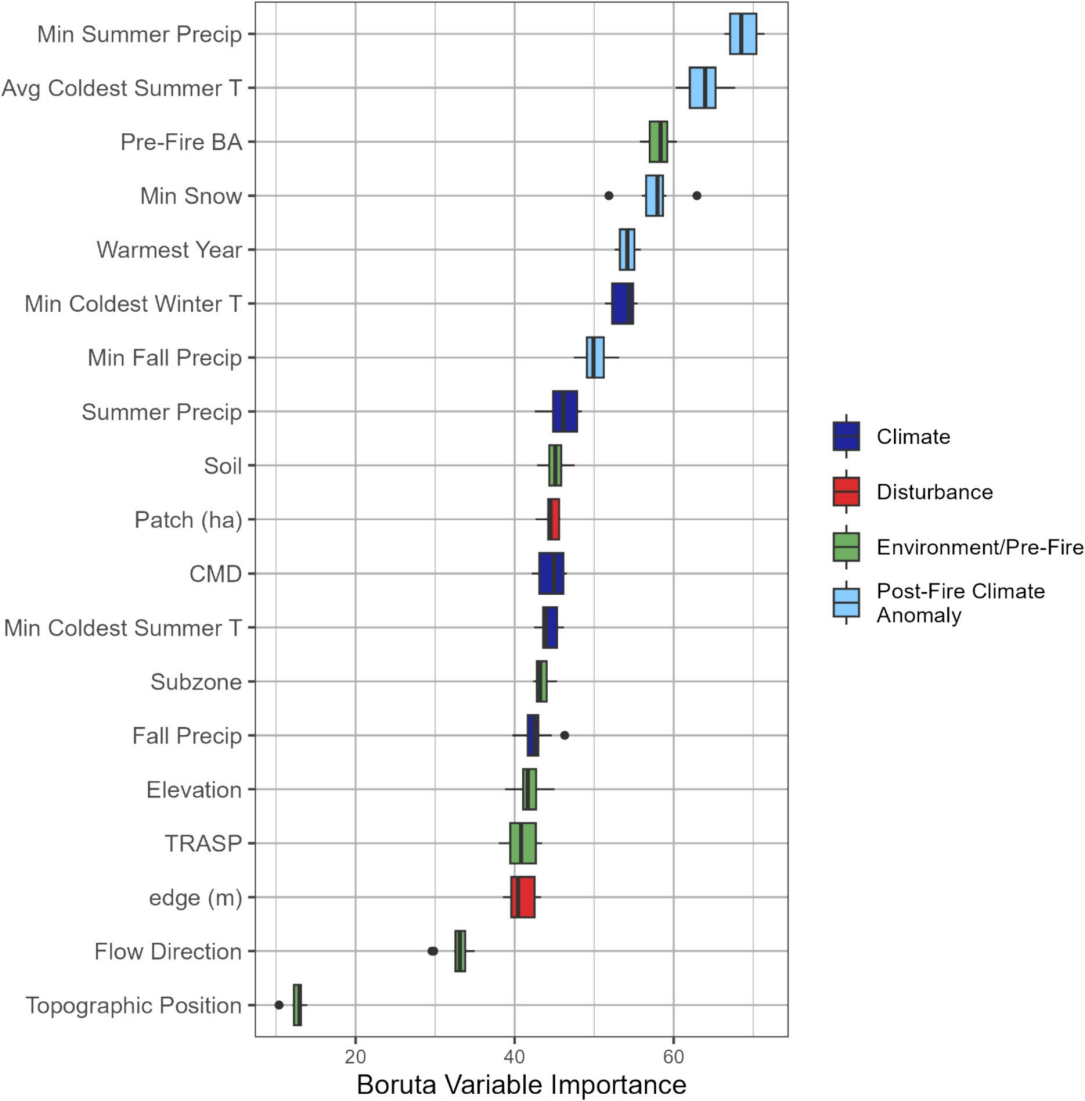
Boxplots for variable importance for random forest built for the global model with all driver Categories. Distribution is the Boruta variable importance for 500 random forest trees. Importance range 0–100, where 0 is no impact

In the global model, anomalously cold and dry summers were the most important predictors of observed groupings (minimum summer precipitation and average coldest summer temperature, Fig. 5). Annual climate anomalies, such as the warmest year and minimum summer temperature were also important. In the Boruta algorithm, when these metrics were removed, the explanatory power of the model decreased by 54 and 58% respectively. Typical climate conditions, including minimum winter temperature, or average fall and summer precipitation, generally followed climate anomalies in overall importance. Fire impacts (patch size and distance-to-edge) and site-level/environmental conditions (soil type, subzone, TRASP, and elevation) had similar relative importance values (41–45%). Topographic position had a notably lower importance than other variables (relative importance = 12%).

## The relative effects of dominant ecological drivers on structural groupings

**Fig. 6:**
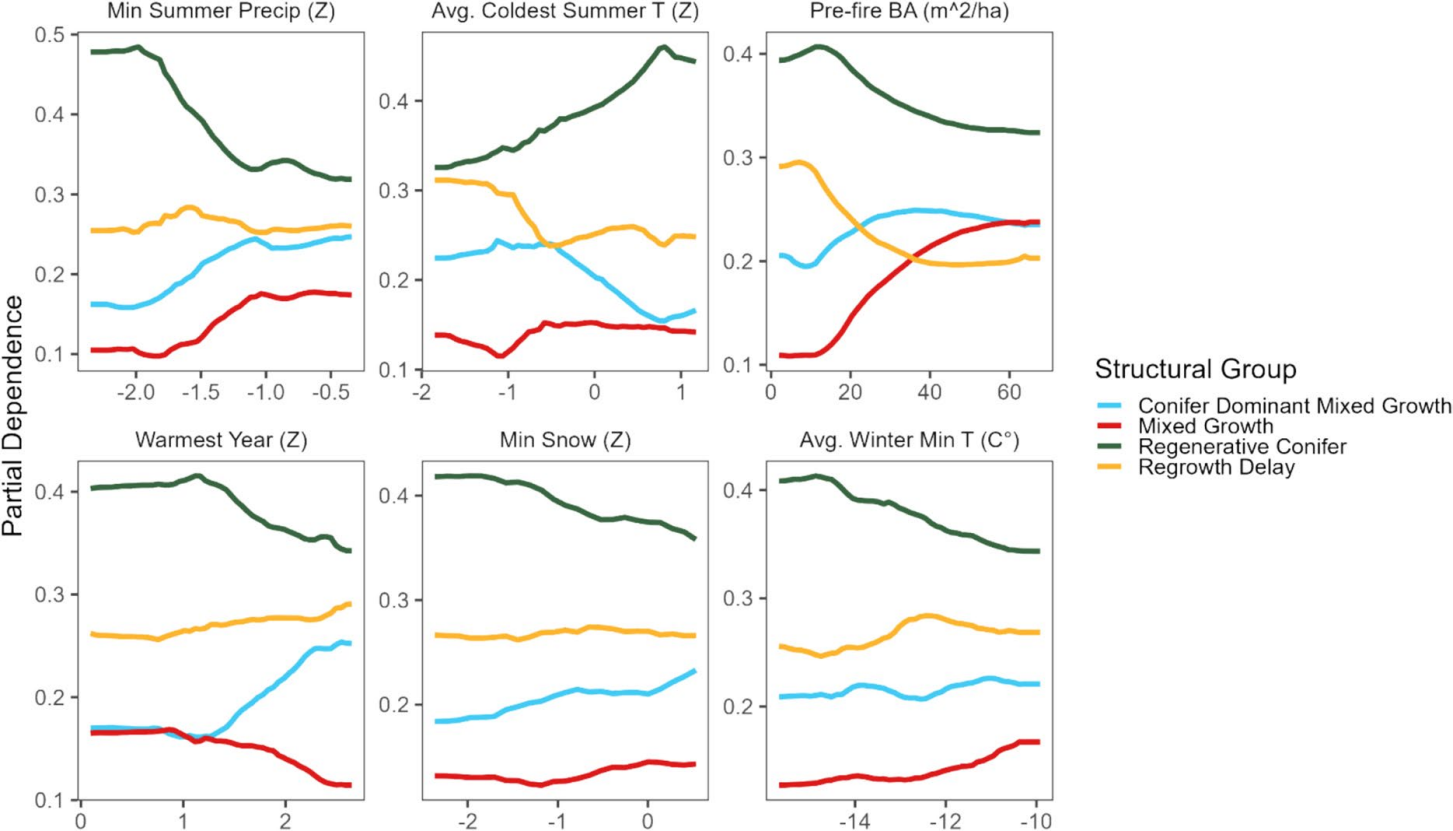
Partial dependence plots for the top 6 most important drivers from global random forest (19 selected drivers). The X-axis represents predictor variable range, and the y-axis is likelihood for structural with all other variables in the random forest held constant. Variables are listed in decreasing importance for random forest. Labels above plots note the units for each driver. “Z” is the z-score standardized value based on climate data 1980–2010

We used the global model to compare the marginal effects of the six most important drivers. Partial dependence plots demonstrated structural group likelihoods were associated with different ecological driver values (Fig. 6). In some cases, only one structural group had different responses to an ecological driver, such as minimum summer precipitation. Regenerative conifer was the only structural group not negatively affected by minimum summer precipitation (Fig. 6). Comparatively, coniferous dominant mixed growth was positively associated with warmest year. However, mixed growth was negatively related to warm post-fire years but positively associated with a lack of dry summers. Additionally, minimum snow deposition had a slightly positive effect on regenerative conifer, and a slightly negative effect on coniferous dominant mixed growth.

Pre-fire BA had variable effects on structural groups. Generally, groups with a higher proportion of deciduous stems after fire were positively associated with greater pre-fire BA (both mixed growth and coniferous dominant mixed growth had higher pre-fire BA).

## Differences in pre-fire conditions and fire impacts across structural groups

**Fig. 7:**
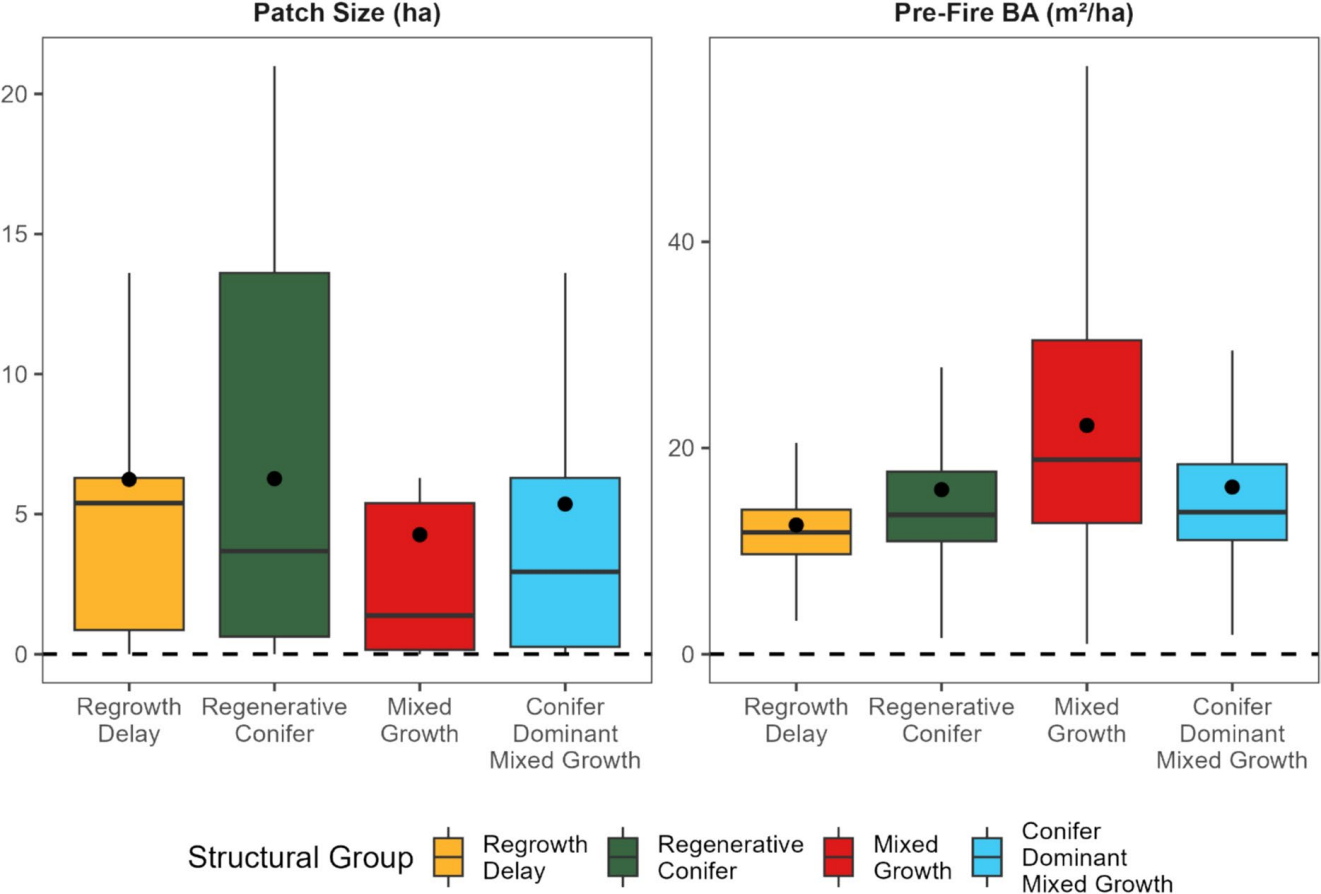
Boxplots of structural groupings for patch size calculated from NTEMS dNBR data (left) and pre-fire mean (*n* = 5 years) BA in m2 / ha (right). Boxes are colored by structural group. Black lines are median structural value with boxes spanning the interquartile range and 95 % confidence values. Black points are the mean value for each structural group

The most important drivers for fire impacts and pre-fire conditions categories in the global RF model were pre-fire BA and burned patch size. In the global RF, pre-fire BA was the third most important predictor (Fig. 5). Pre-fire BA estimates were highest in mixed growth (average BA = 24.60 m^2^/ha), while lowest pre-fire BA estimates were in regrowth delay areas (12.5 m^2^/ha). The lower significance of pre-fire BA in the overall RF (Fig. 5) is likely because pre-fire BA was similar between conifer dominant mixed growth and regenerative conifer (Fig. 7 pre-fire BA = 16.2 vs 16.0 m^2^/ha respectively).

Patch size was the most important variable for fire impacts (compared to distance-to-edge). Similar to pre-fire BA, conifer dominant mixed growth and regenerative conifer had similar patch sizes (Fig. 7, patch size = 5.36 vs 6.40 ha). Comparatively, mixed growth had the smallest patch sizes (average patch size = 4.30 ha) and the highest pre-fire BA estimates (average BA = 24.60 m^2^/ha). Finally, regrowth delay had the highest average patch size (average = 6.2 ha).

## Discussion

Post-fire trends and patterns of structural recovery were strongly impacted by a *s*ingle year of cold or dry summers within the first five years after a fire (Fig. 6). We found eight unique spectral responses that characterized four groups of structural forest recovery, each with different ratios of coniferous and deciduous cover, stem densities, and rates of conifer establishment. Of the four categories of recovery drivers (environment, climate variables, post-fire climate anomalies, fire impacts and pre-fire conditions), post-fire climate anomalies best predicted structural recovery (Fig. 5). Specifically, anomalous cold and dry periods impacted regeneration timing and the ratio of deciduous stems; areas of delayed coniferous regeneration experienced particularly cold summers. In contrast, areas with a higher proportion of deciduous cover (coniferous dominant mixed recovery), were less likely to have cold summers and generally had summers with greater precipitation. Our approach, fusing spectral with lidar data to characterize structural forest recovery, addresses the important need for understanding recovery across the vast burned environments of the sub-boreal.

## Early spectral responses described longer-term differences in structural recovery

When the eight spectral clusters were merged into four unique groupings of structural recovery, the structural groups remained unique in spectral responses (Fig S3). Thus, these early spectral dynamics characterized longer-term differences in structural recovery, specifically differences in the rate of coniferous establishment, proportion of deciduous trees, and stem densities. Our research builds on the connection between unique post-fire spectral responses and differences in structural recovery (Guz et al. 2022; Kiel and Turner 2022; Smith-Tripp et al. 2024b), but as structural groupings did not directly mirror those identified by Smith-Tripp et al. (2024b) our findings raise important considerations when implementing a space-for-time study using spectral trends.

There are similarities between our four structural recovery groups and recovery patterns and trends observed in other conifer forests (Littlefield 2019; Kiel and Turner 2022; Menick et al. 2024). Additional forest structural data from lidar acquisitions supplemental to the dataset of Smith-Tripp et al. (2024b) clarified trends and patterns of recovery. For example, Smith-Tripp (2024b) identified some areas with remaining residual canopy and delayed regrowth – but the sample size for these areas was low (25 pixels). Additional sampling (3,778 pixels) confirmed these areas, classed as regrowth delay, had high ground cover and stem densities five years post-fire, which may be from residual canopy, as well as delayed regrowth. 8–12 years post-fire regrowth delay had relatively low stem densities and a high proportion of bare ground 8–12 years post-fire, supporting slow conifer establishment. The slow conifer establishment rate of regrowth delay has been observed elsewhere in Western NA (Littlefield 2019; Menick et al. 2024; Kiel et al. 2025). All these studies support that new coniferous stems can take 10–20 years to establish and/or detect.

Interestingly, the group with the highest conifer stem density in the early post-fire (+ 5) years, was mixed growth. However, high stem densities in mixed growth could also reflect residual canopy– a response also noted by Smith-Tripp et al. (2024b). The loss of residual canopy (common in the first decade post-fire; Bolton et al. 2015; Frazier et al. 2018) would also explain the drop in stem-density in years 8–9. Research in conifer forests often associates high-severity fire with post-fire dominance of shrub or aspens (Lee 2004; Paudel and Markwith 2023), but we found burn severity was not indicative of a deciduous response. The group with the highest burn severity (conifer dominant mixed growth) had the highest proportion of deciduous cover in years 5–7 (Table S2) but maintained a conifer dominant composition throughout the study period.

Future research in dry coniferous forests should be cautious to associate “fast” spectral recovery with deciduous recovery. In our research the group with the highest deciduous component, mixed growth, also had the highest spectral recovery for NBR. Yet NDVI spectral recovery rates were higher for regenerative conifer, which had the highest proportion of conifers and greatest stem density. Thus, in dry coniferous forests, a faster spectral recovery rates for indices such as NBR or NDVI is not indicative of a greater deciduous abundance. This conclusion aligns with findings of Celebrezze et al. (2024). They found areas of “fast” and “slow” spectral recovery (both NBR and NDVI) distinguished shrubs and conifers from grass but could not distinguish shrubs among conifers. Notably, we found “slow” spectral recovery rates were consistently associated with regrowth delay, likely a result of a high proportion of bare ground in the early post-fire years. In conifer forests, spectral recovery rates are then best associated with a description of general vegetative recovery.

By including multiple spectral indices our spectral groups successfully separated mixed growth responses from areas of regenerative conifer. Spectral recovery rates for mixed growth did not differ from rates for the regenerative conifer group based on a single index, but multiple indices improved separation. Past research in dry conifer forests has struggled to differentiate deciduous from conifers using spectral data (Blanco-Rodríguez et al. 2023). To address these difficulties, Blanco-Rodríguez et al. (2023) suggest monitoring approaches that combine climate, site-level conditions, and spectral data. Importantly, integrating environmental conditions increases the accuracy of recovery monitoring from satellite data (Pérez-Cabello et al. 2021), but our approach relying on spectral data alone helps identify areas of similar structural recovery despite differences in environmental conditions. For example, regrowth delay was predominant in cold-dry sites, but regrowth delay was also located in areas that were normally wet and productive (Fig. 6). Including environmental conditions in classification may fail to identify areas unexpected to have regrowth delay, such as normally wet and productive regions. In all cases, it is important to validate spectrally identified recovery with field data because spectral responses, while useful to identify overall rates of vegetation growth, cannot universally distinguish shrubs and trees.

Our space-for-time approach lacks precision to compare group temporal trends. Consider the regrowth delay class, which had extremely high stem densities 15–16 years post-fire, and lower stem densities in year 21. The drop in stem density between years 15 and 21 could signify the start of stem-exclusion (Bartels et al. 2016), but it could also be a result of site differences not considered in our space-for-time approach. Fortunately, BC is currently conducting a province-wide lidar acquisition (White et al. 2025). This acquisition will provide high-quality, spatially continuous lidar data, allowing future work to test how site-level differences influence recovery, such as the onset of stem exclusion. In addition to capitalizing on lidar data, future work should incorporate additional information on tree species composition and structure using remote sensing and/or field data (White et al. 2023). This information could clarify how observed structural recovery aligns or does not align with management goals and historic ecosystem composition (Johnstone et al. 2016). For example, mixed growth responses may align with pre-fire composition (Jorgensen et al. 2023) and/or historic deciduous forest composition altered by 20th-century forest management (Brookes et al. 2021; Baron et al. 2022).

## Structural group variability was associated with anomalous post-fire climate

We found that structural recovery was best predicted by anomalous post-fire temperatures and precipitation, echoing research findings in coniferous dry Western US forests (Table S3, Young et al. 2019; Guz et al. 2021). Prior research suggests that drought conditions, measured via climate moisture deficit (CMD), negatively impact coniferous recruitment (Talucci et al. 2019; Stevens-Rumann et al. 2022; Davis et al. 2023). In our study, colder dry summers were more important than typical CMD. The decreased importance of CMD may be because lodgepole pine, the dominant conifer of the study area, is less impacted by drought than other conifers (Harvey et al. 2016). Further, lodgepole pine establishment and growth can be limited at low temperatures (Hansen and Turner 2019).

The positive association of a single particularly cold summer and the slow establishment rates of regrowth delay suggests lodgepole pine establishment in sub-boreal may be temperature limited. The relationship between delayed lodgepole pine establishment and lower temperatures has also been found in subalpine environments in southern Colorado (Guz et al. 2021) and post-fire forests of the Greater Yellowstone ecosystem (Hansen and Turner 2019). This delayed establishment was also noted in regions impacted by MBP in the mid-2000s (Dhar et al. 2016a). In our study area, the average summer temperature from 1980 to 2010 was 12.7 ˚C, below the 14 ˚C threshold identified by Hansen and Turner (2019) as the temperature where lodgepole pine recruitment is limited more by temperature than moisture.

The two structural groups with a higher deciduous proportion had different responses to extreme post-fire temperatures and precipitation (Fig. 6). For example, mixed growth was negatively impacted by dry summers and anomalously warm years. Conversely, conifer dominant mixed growth was positively associated with anomalously warm average annual temperatures. These differences could reflect the availability of underground resources, as resprouting deciduous species (both aspen and shrubs) can rely on surviving underground resources to buffer impacts of extreme climate (Young et al. 2019; Johnstone et al. 2020). Importantly, these unburned below-ground resources are inaccurately captured with satellite-estimated burn severity (Loboda et al. 2013). Satellite measures of burn severity characterize remaining above-ground vegetation post-fire (Miller and Thode 2007; Frolking et al. 2009). To better understand the relationship between climate extremes and deciduous regeneration post-fire, recovery models should also consider the remaining soil organic material that facilitates post-fire deciduous resprouting (Shenoy et al. 2011), delays competing conifer establishment (Stark et al. 2006), and enables deciduous resprouts to endure climate extremes (Young et al. 2019).

The association between lagged coniferous establishment and cold summers in our studies' sub-boreal environment has important implications given observed and projected warming in BC (MacKenzie and Mahony 2021). While increasing temperatures may decrease areas with delayed regrowth, drought conditions could accompany rising temperatures. Prolonged droughts can independently cause regeneration lag (Guz et al. 2021) or even coniferous regeneration failure (Stevens-Rumann et al. 2022). As multi-annual climate conditions have also been linked to differences in coniferous recovery (Kemp et al. 2019; Shuang and Christopher 2012), future research should consider the impact of prolonged (> 1 year) droughts and mean climate conditions multiple years post-fire.

## Site-specific conditions had a minimal impact on individual structural groupings

We found pre-fire basal area (BA) was the third most important driver across all structural groupings. This finding may reflect the similar pre-fire BA between regenerative conifer and conifer dominant mixed growth groups. Research in the boreal forest supported that greater pre-fire BA was associated with slower NBR recovery rates (White et al. 2023). The authors found that areas that had not yet achieved spectral recovery (80% of the pre-fire NBR value) by the end of the time series had a higher pre-fire BA. However, in the study of White et al. (2023) sites with a higher pre-fire BA also had a higher pre-fire NBR, meaning that sites required a greater magnitude of change to reach pre-fire NBR values. In our research, higher pre-fire BA were generally related to areas with greater rates of spectral recovery. Yet, we emphasize that our spectral recovery metric (rate of recovery) differs from the years-to-recovery metric of White et al. (2023). In our sub-boreal study region, productivity is soil nutrient-limited (DeLong et al. 2003). While we did not include soil nutrients in models, areas of high soil nutrient density may drive spatial clusters of high pre-fire BA. Thus, interactions among ecological drivers, such as pre-fire BA and soil nutrients, may cause differences in recovery drivers across regions.

Contrary to recent research, we found a minimal impact of elevation on recovery trends. White et al. (2023) found a greater proportion of high elevations field plots had not yet spectrally recovered by the end of the time series (25 years post-fire). Similarly, Kiel and Turner (2022) found elevation had the largest impact on spectral recovery and stem densities 30 years post fire, followed by slope and distance-to-seed. However, Kiel and Turner (2022) did not consider post-fire climate conditions and White et al. (2023) did not directly test the impact of elevation against climate conditions. Thus, differences between our findings and those Kiel and Turner (2022) as well as White et al. (2023) suggest that the importance of recovery drivers likely depends on which drivers are considered, how recovery is defined, and which spectral metrics are used for recovery assessments. Additionally, Kiel and Turner (2022) measured recovery of a single fire year (1988). This suggests that recovery from a single fire year, which experiences the same after fire conditions, may be more driven by site-level differences such as elevation, but for fires that occur in different years, recovery may be more controlled by differences in post-fire climate conditions across years.

## Post-fire climate and the need for adaptive post-fire management

The need for post-fire management actions is often based on assumed impacts of recovery drivers (North et al. 2019). For example, Larson et al. (2022) provide a post-fire planting framework that prioritizes planting in areas of high burn severity, unfavorable site composition, and projected harsh future climates. In their framework, harsh future climates are based on projected, rather than measured, climate conditions (Larson et al. 2022). Given the link with coniferous establishment and measured post-fire climate observed in our research and elsewhere (Littlefield 2019), we suggest post-fire management should adapt to measured post-fire climates.

Post-fire management must adapt to observed climate conditions to preserve key ecosystem services. For example, vegetation mitigates the risk of post-fire landslide (Hope et al. 2015). In the context of our work, regrowth delay had the greatest amount of bare ground 8–9 years after fire. Regrowth delay was more likely in areas with particularly cold or dry summers, suggesting that cold and dry summers may promote a longer period of barren land. In some cases, planting deciduous species would improve the rate of overall vegetation recovery, which promotes slope stability and decreases future fire risk (Wang et al. 2016).

Frameworks prioritizing post-fire management responses based on site conditions and projected climate, such as Larson et al. (2022), are crucial for forest management. However, given the importance of anomalous post-fire climate found in our work and elsewhere in the literature (Stevens‐Rumann et al. 2018; Davis et al. 2023), frameworks should be iterative, with an initial response developed immediately post-fire and later adaptation based on post-fire climate conditions. Swanson et al. (2023) argue that if post-fire climate conditions promote strong coniferous growth that results in dense young forest, then additional management intervention may be necessary to decrease fire risk. Our approach to quantifying recovery across the entire burned landscape, helps forest managers prioritize action areas, including regions with heavy post-fire fuel loads and/or barren landscapes prone to landslides (Larson et al. 2022; Lau 2022; Swanson et al. 2023; Davis et al. 2024).

## Conclusions

Using a combination of satellite and RPA lidar data, we found strong post-fire conifer recruitment across areas of BC’s sub-boreal forest that were burned by high severity wildfires. Additionally, we found that early (i.e., within 5 years’ post-fire) satellite-measured spectral recovery indicated forest groups with different patterns and rates of structural forest recovery. Post-fire anomalous summertime climates and pre-fire BA best predicted these structural recovery groups. Specifically, abnormally cold and dry summers delayed conifer establishment, while warm years and/or higher pre-fire BA increased post-fire deciduous recruitment. Effective forest management in a rapidly changing climate with intensified fire regimes requires an understanding of what drives post-fire forest recovery. To extend the use of the work presented herein, future research should investigate how longer-term climate conditions (e.g., drought conditions over multiple years) or better soil burn severity classification (which captures available below-ground resources) improve the detection of post-fire structural recovery. Ultimately, our structural recovery groupings and associated ecological drivers help forest managers prioritize action and non-action areas. Action areas include regions of regrowth delay, where barren landscapes could promote slope instability, while non-action areas, such as regions of conifer-dominant mixed growth, restore the historic forest composition and mitigate future fire risk. Importantly, the likelihood of an action area, such as regrowth delay, is associated with post-fire climate extremes. Thus, monitoring and management should capture the initial fire event and adapt as conditions evolve during the crucial early recovery period.

## Supplementary Information

Below is the link to the electronic supplementary material.

Supplementary file1 (DOCX 4124 KB)

## Data Availability

No datasets were generated or analysed during the current study.
