# Supplementary material for: Post-fire structural forest recovery associated with climate extremes in dry sub-boreal forests
Source: Landsc Ecol. 2025 Dec 1;41(1):14. doi: 10.1007/s10980-025-02266-y (PMC12779702; doi:10.1007/s10980-025-02266-y)
Supplement: Supplementary file 1 — Supplementary file1 (DOCX 4124 KB) [file 10980_2025_2266_MOESM1_ESM.docx]

**Supplementary Information**

**Post-Fire Structural Forest Recovery Associated with Climate Extremes in Dry Sub-Boreal Forests**

Sarah Smith-Tripp^1*^, Nicholas Coops^1^, Christopher Mulverhill^1^, Joanne White^2^, Sarah Gergel^3^

^*^ *Corresponding Author:* [*sarahsmith.tripp@alumni.ubc.ca*](mailto:sarahsmith.tripp@alumni.ubc.ca) *ORCID:* [*https://orcid.org/0000-0003-0347-7165*](https://orcid.org/0000-0003-0347-7165)

^1^ Faculty of Forestry, University of British Columbia, 2424 Main Mall, Vancouver BC, Canada

^2^ Canadian Forest Service, (Pacific Forestry Centre), Natural Resources Canada, 506 West Burnside Road, Victoria, BC V8Z 1M5, Canada

^3^ Department of Forest and Conservation Sciences, Forest Sciences Center, University of British Columbia, 3024-2424 Main Mall, Vancouver V6T 1Z4, British Columbia, Canada

1. Structural Model Development

We used an area-based approach (ABA) to create spatially continuous estimates of BA, stem density, the ratio of conifer and deciduous cover (composition), and the fraction of the landscape with bare ground in the Lidar acquisitions. We created these estimates using area-based estimates from a series of ground plots 17-m radius (900 m^2^) deployed at each flight location (total number of field plots = 32). Ground plot locations were designed to be representative of the forest structure within the flight areas. At each plot, we estimate the proportion of ground cover for bare ground, deciduous (including shrubs and trees), and conifer cover. We counted all stems at DBH (1.3 m) and measured all stems with a DBH greater than 4 cm. We highlight that this DBH cutoff, while lower than that of some other post-disturbance studies (Mulverhill et al. 2018) resulted in relatively low BA across plots. To address the low BA observed in many of the sample plots, we supplemented with additional lidar data from an unburned forest type (n=4), but note that the DBH cutoff likely decreased the overall BA of plots.

Area-based estimates were created using the linear relationships between ground plots and Lidar point cloud derived metrics including basic, dispersion, L-moments, and percentile metrics calculated with the LidRmetrics package (Roussel et al. 2021). For composition and bare ground models we also added a random effect of disturbance year or acquisition as these models were sensitive to the season of data collection and differences in the lidar acquisition parameters. Final model accuracies based on k-fold evaluations of 100 models. Models relied on distributions and the formulas available in the *mgcv* and *glmmTMB packages* (Wood 2021; Brooks et al. 2022). All models were assessed for normality and heteroskedasticity. Model values were transformed when necessary and final models were weighted necessary. Model accuracy and coefficients are noted in Table S1. When models were applied at the landscape-scale, we excluded areas where model estimates were greater than those observed in the field.

The final linear models were applied across all lidar acquisitions to produce 1300 ha of post-fire structural estimates for the four different structural measures (basal area, the ratio of deciduous to conifer, bare-ground, and stem densities). These structural estimates were later used to merge spectral clusters.

*Table S1: Model coefficients and model type for the four modeled structure variables. Model type includes the model family. Df = degrees of freedom. Measure of fit (R^2^ or AUC) is included for each model.*

| Model | Model Type | Df | Beta | Estimate | Standard Error of estimate | R^2^ |
| --- | --- | --- | --- | --- | --- | --- |
| Basal Area | Binomial (log-link) |  | (Intercept) | -0.22 | 0.76 | AUC = 0.79 |
|  |  | 29 | L2 | 35.65 | 17.30 |  |
|  |  |  | L3 | -49.52 | 24.23 |  |
|  |  |  | (Intercept) | 8.76 | 1.67 | 0.92 |
|  |  |  | pzabovemean | -0.21 | 0.04 |  |
|  |  |  | L3 | 2.44 | 0.78 |  |
| Basal Area | Gamma log-link | 17 | L4 | -3.27 | 1.00 |  |
|  |  |  | Lkurt | -11.61 | 2.02 |  |
|  |  |  | (Intercept) | 5.95 | 2.23 |  |
|  | Beta AR log-link |  | (Intercept) | -11.88 | 7.14 |  |
| Coniferous:Deciduous |  |  | Pzabovemean | 0.11 | 0.09 | 0.46 |
|  |  |  | CRR | 21.32 | 9.24 |  |
|  |  |  | Lkurt | 10.65 | 4.18 |  |
|  |  |  | Lcoefvar | 3.23 | 5.35 |  |
|  |  |  | betadjustment | 0.54 | 0.64 |  |
|  |  |  | theta | 0.68 | 0.86 |  |
|  |  |  | Disturbance Year |  | 1.50 |  |
|  |  |  | **Random Effect* |  |  |  |
|  |  |  | (Intercept) | 5.94 | 2.10 | 0.59 |
| % Bare Ground | Beta AR log-link |  | Zcv | 0.00 | 0.00 |  |
|  |  |  | Zkurt | 0.00 | 0.00 |  |
|  |  |  | Pzabovemean | -0.08 | 0.03 |  |
|  |  | 11 | Pzabove2 | 0.05 | 0.02 |  |
|  |  |  | CRR | 3.84 | 2.12 |  |
|  |  |  | Lkurt | -3.81 | 1.71 |  |
|  |  |  | Vertical Complexity Index | -6.00 | 1.10 |  |
|  |  |  | Lcoefvar | -1.92 | 1.77 |  |
|  |  |  | Sample Plot* |  |  |  |
|  |  |  | **Random Effect* |  |  |  |
| Stem counts | Zero inflated negative binomial |  | (Intercept) | 2.45 | 1.57 |  |
|  |  |  | Lkurt | -3.30 | 3.09 |  |
|  |  | 28 | (intercept) | -11.88 | 0.44 | 0.69 |
|  |  |  | Ziqr | 0.11 | 0.41 |  |
|  |  |  | CRR | 21.32 | 4.00 |  |
|  |  |  | Ttops | 10.65 | 0.00 |  |
|  |  |  | VCI | 3.23 | 2.31 |  |

1. Structural Groupings


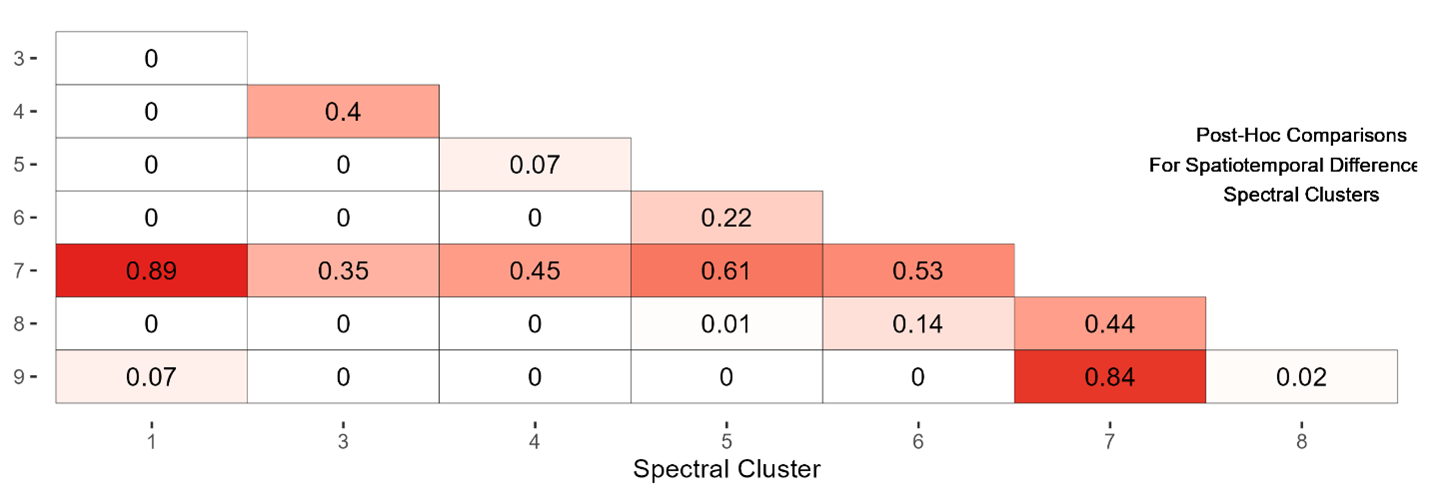


1. Post-hoc description of differences in spectral clusters based on structural variables input into MANOVA analysis. Spectral grouping combined spectral clusters based on similarity. Values are adjusted using the p-holm method for non-parametric data. Cluster 7 was similar across all clusters, but was combined with cluster 1 because this had the highest degree of similarity (p = 0.89). Groupings are as follows: Mixed Growth = cluster 1 and cluster 7, regrowth delay = cluster 3 and cluster 4, Regenerative Conifer = Cluster 5, 6, and 8, Coniferous dominant mixed growth = cluster 9.


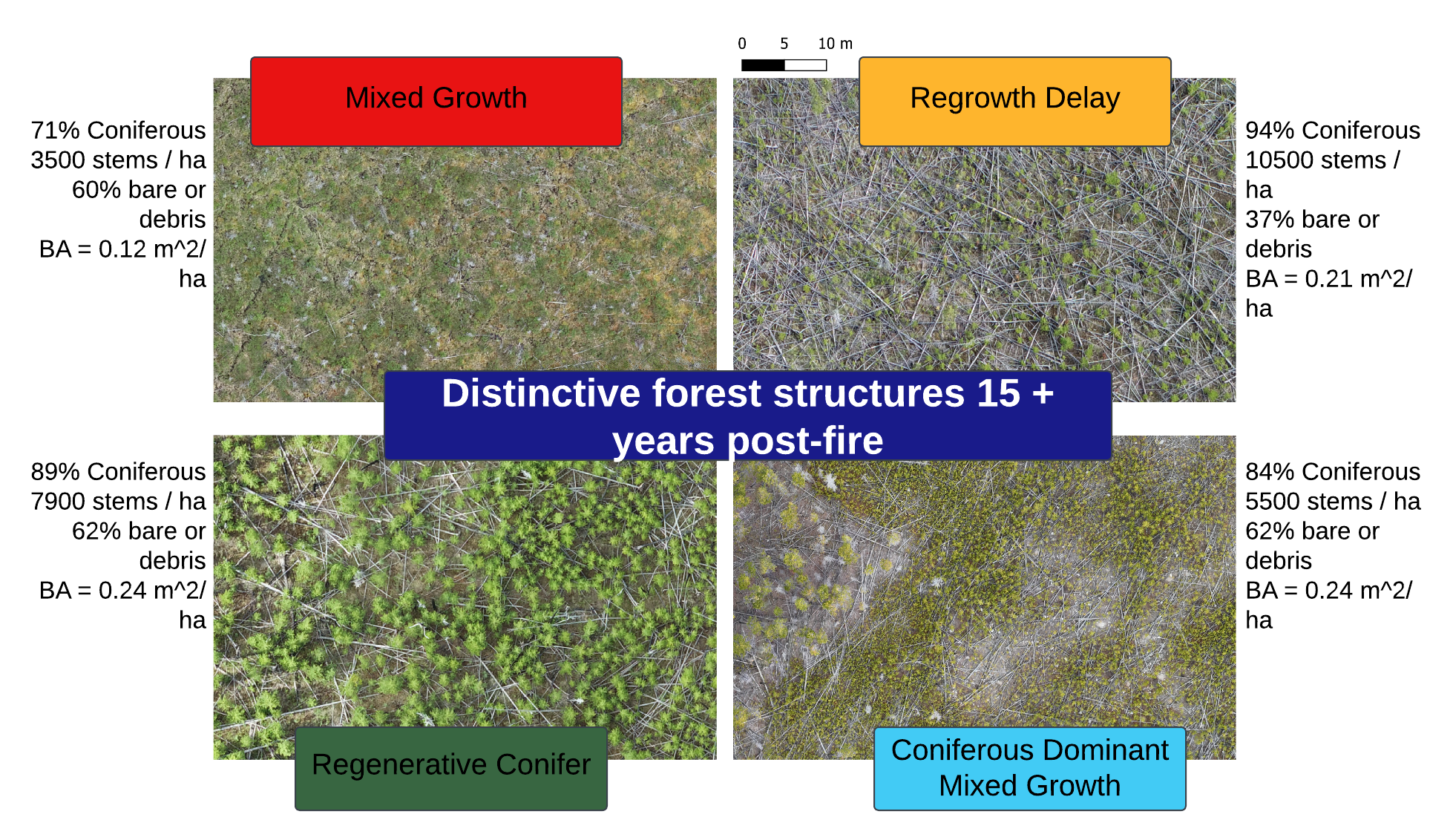


1. Remotely Piloted Aircraft orthophotos of structural recovery groups established from spectral clustering at 15-16 years post-fire. Values note the average values for each structural group 15-16 years post-fire. All images are at the same scale.

Table S2 Average values for each by structural group (+/- standard deviation). Values are colored by the scale by the minimum and maximum across all years.


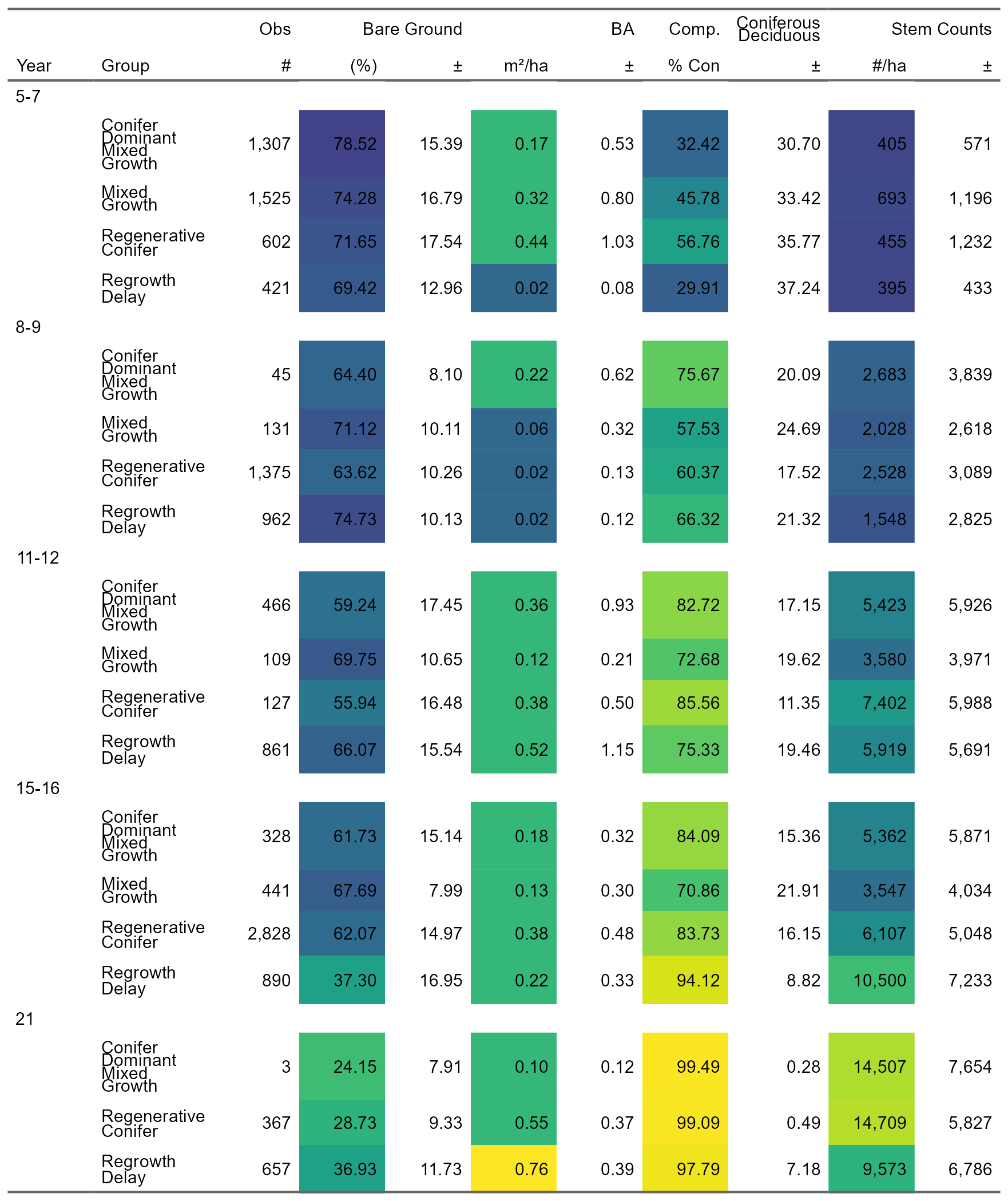


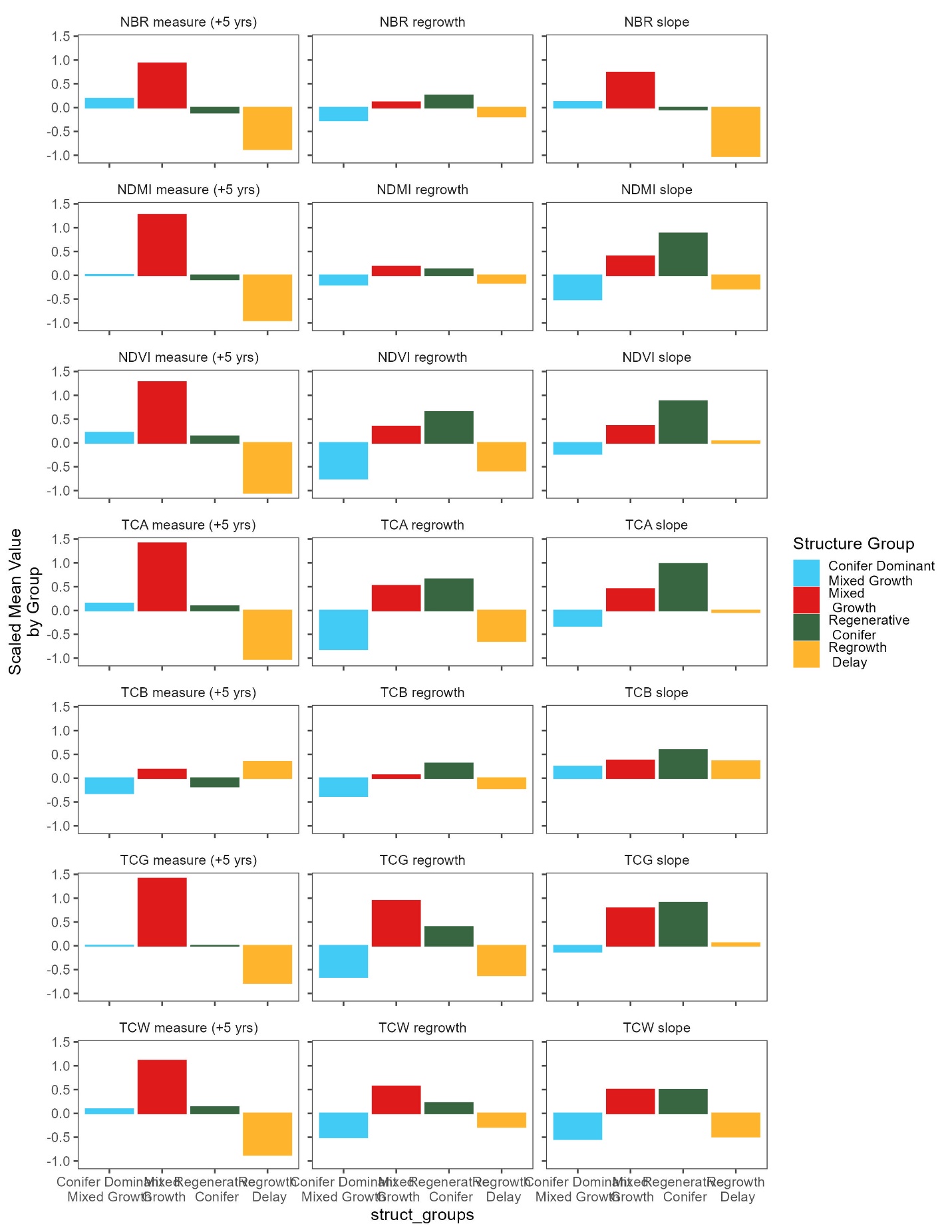


1. Average values of spectral metrics included in clustering for structural groups. Values are the normalized scaling applied before metrics are input into k-means clustering algorithm.
2. Random Forest Models

| Type | Ovr.Accuracy |  | Variable Importance |
| --- | --- | --- | --- |
| environmental | 0.54 | Elevation (m) | 0.04 |
|  |  | Soil | 0.03 |
|  |  | TRASP | 0.02 |
|  |  | Flow Direction | 0.02 |
|  |  | Topographic Position | 0.01 |
| prefire | 0.66 | Subzone | 0.12 |
|  |  | Patch (ha) | 0.11 |
|  |  | Pre-Fire BA | 0.05 |
|  |  | edge (m) | 0.02 |
|  |  | Pre-Fire Species | 0.01 |
|  |  | Pre-Fir Land Cover | 0.00 |
| climate | 0.83 | Winter Min T | 0.18 |
|  |  | Summer Min T | 0.18 |
|  |  | Fall Precip | 0.17 |
|  |  | Summer Precip | 0.17 |
|  |  | Avg Precip | 0.16 |
|  |  | CMD | 0.15 |
|  |  | Spring Precip | 0.15 |
|  |  | Avg Summer T | 0.14 |
|  |  | Summer Max T | 0.14 |
|  |  | PAS | 0.14 |
|  |  | MAT | 0.14 |
| anomaly | 0.84 | Min Summer Precip | 0.18 |
|  |  | Warmest Year | 0.17 |
|  |  | Min Fall Precip | 0.16 |
|  |  | Avg Coldest Summer T | 0.15 |
|  |  | Min Snow | 0.15 |
|  |  | Min Summer T | 0.15 |
|  |  | Min Annual Year | 0.14 |
|  |  | Max Summer T | 0.13 |
|  |  | Min Coldest Winter T | 0.13 |
|  |  | Hottest Summer T | 0.13 |
|  |  | Max Warmest Winter T | 0.13 |
|  |  | Driest Year | 0.12 |
| overall | 0.84 | Min Summer Precip | 0.69 |
|  |  | Avg Coldest Summer T | 0.64 |
|  |  | Pre-Fire BA | 0.58 |
|  |  | Min Snow | 0.58 |
|  |  | Warmest Year | 0.54 |
|  |  | Min Coldest Winter T | 0.54 |
| Type | Ovr.Accuracy |  | Variable Importance |
| Overall (cont) |  | Min Fall Precip | 0.50 |
|  |  | Summer Precip | 0.46 |
|  |  | Soil | 0.45 |
|  |  | Patch (ha) | 0.45 |
|  |  | CMD | 0.45 |
|  |  | Min Coldest Summer T | 0.44 |
|  |  | Subzone | 0.43 |
|  |  | Fall Precip | 0.43 |
|  |  | Elevation (m) | 0.42 |
|  |  | TRASP | 0.41 |
|  |  | edge (m) | 0.41 |
|  |  | Flow Direction | 0.33 |
|  |  | Topographic Position | 0.12 |

*Table S3: Random forests built for different driver types. For each random forest the overall accuracy, variables, and associated variable importance is noted. The overall random forest selected no more than five variables of each recovery driver type and no driver with a variable importance < 0.01*


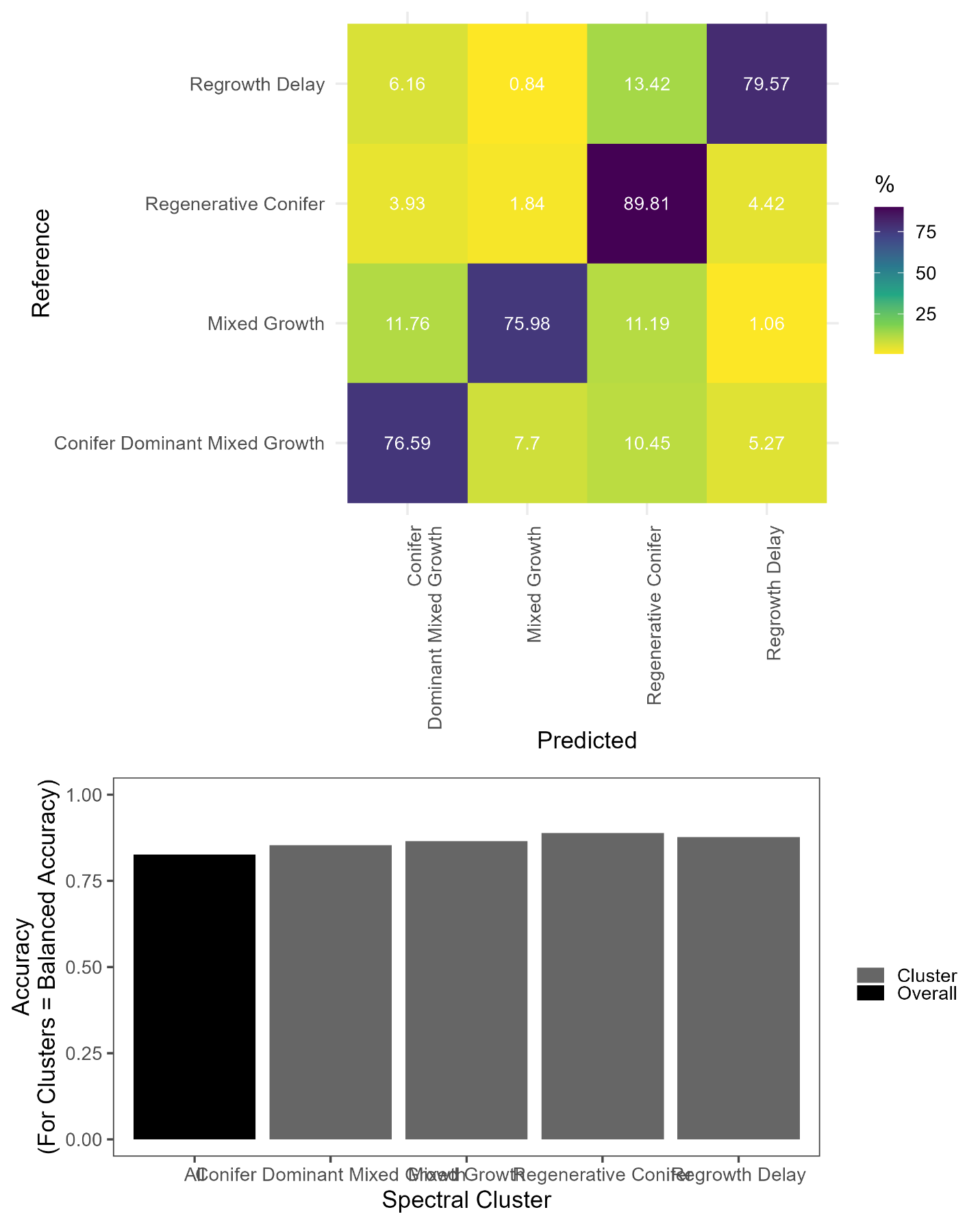


1. Confusion matrix for overall random forest model classifying structural recovery from validation data (n = 14440) colored by the % of correctly classified pixels.
